# Recent advances in understanding maternal perinatal mood disorders

**DOI:** 10.12688/f1000research.10560.1

**Published:** 2017-06-15

**Authors:** Thalia Robakis, Eugenia Jernick, Katherine Williams

**Affiliations:** 1 Department of Psychiatry and Behavioral Sciences, Stanford University, Stanford, CA, USA

**Keywords:** perinatal, psychotropic medication, maternal mental illness, mood disruption

## Abstract

The study of perinatal mental health (mental health during pregnancy and postpartum) is a complex field of study that is of major importance both for the mental and physical health of new mothers and for the neurobehavioral development and long-term functioning of the children they bear. In this review, we cover the most recent additions to this rapidly evolving field. Notable advances include further illumination of the epidemiological patterns and clinical manifestations of perinatal mood disruption; new efficacy data on treatment and prevention; clarifications of the respective contributions of maternal mental illness and psychotropic medication to outcomes of pregnancy, birth, and child development; and updated expert guidelines for screening.

## Introduction

The most recent advances in the study of perinatal mental health fall into several categories. We have first addressed updates to our understanding of the epidemiological patterns and clinical manifestations of perinatal mood disruption. Next, we have discussed updates to expert guidelines for screening. We cover critical recent additions to the ongoing conversation about treatment efficacy and about the relative contributions of maternal mental illness versus psychotropic medications to obstetric, neonatal, and childhood outcomes.

## Part I: Advances in understanding epidemiology, phenomenology, and pathophysiology of perinatal mood disruption

Scholarship in recent years has added important insights to our understanding of the landscape of perinatal mental health. Past work had revealed major roles for mood disorder history (particularly a history of the diagnosis of bipolar disorder, or BD), poor social supports, and a history of maltreatment ^1, 2^. Recent research has provided new information about the role of bipolar diathesis, personality factors, anxiety disorders, and obstetrical complications in the risk for postpartum depression.

Although it has been known for some time that BD confers a greatly increased risk of mood disruption in the perinatal period ^3, 4^, evidence recently has accumulated that many women initially diagnosed with unipolar postpartum depression in fact suffer from “hidden” BD. Sharma *et al.* ^5^ published the results of a prospective study of 142 postpartum women, demonstrating a rate of diagnostic conversion (from unipolar to bipolar) in the postpartum period of 6.52%, which is 11- to 18-fold higher than diagnostic conversion rates measured in other populations. This study also used validated diagnostic interviews with family members to identify family history of BD and found that conversion from unipolar to bipolar in the first six months postpartum was significantly associated with a family history of BD. This first prospective study of “switch rates” in perinatal mood disorders is extremely important as it guides the clinician to pay special attention to family history of BD and highlights the need to monitor postpartum patients carefully to rule out subtle signs of emerging hypomania and mixed symptoms. It also provides an understanding of the role of BD in treatment resistance in postpartum depression, as Sharma *et al.* ^6^ have previously reported high rates of missed BD in patients referred for treatment-resistant postpartum depression. This recent recognition of high rates of bipolar II disorder in patients diagnosed with postpartum depression is further supported by a study by Mandelli *et al.* ^7^ of women with major depression, bipolar I and II which found that 50% of the women with bipolar II had a history of postpartum depression compared with 27.5% of women with bipolar I and 21.6% of women with major depressive disorder.

Furthermore, in a prospective study by Wisner *et al.* ^8^, 10,000 women were screened with the Edinburgh Postnatal Depression Scale (EPDS) between 4 and 6 weeks postpartum, patients were interviewed by using the Structured Clinical Interview for Diagnostic and Statistical Manual of Mental Disorders IV (SCID), and high rates of BDs (22.6%) were found in women who “screened positive” for postpartum depression (EPDS >10). Notably, high rates of anxiety disorders were also found, as nearly two thirds of patients had comorbid anxiety disorders. Anxiety during pregnancy is now recognized as an important risk factor for postpartum depressive symptoms ^9^ as well as a risk factor for longer time to treatment response in postpartum depression ^10^.

Recognition of the importance of maternal personality factors, particularly insecure attachment style, has also been growing. Robakis *et al.* (2016) (n = 107) ^11^ and Ikeda *et al.* (2016) ^12^ (n = 76) both published the results of prospective studies indicating a major role for antenatally measured attachment insecurity in the risk for postpartum depression. Robakis *et al.* used a longitudinal follow-up design that revealed the importance of maternal attachment style to be specific to the early postpartum period, whereas more classically recognized risk factors such as mood disorder history were of greater importance in predicting depression after the third postpartum month. Personality disorders existing prior to conception were demonstrated to be related to anxious and depressive symptoms in pregnancy, independently of the effect of coexisting mood or anxiety disorders ^13^. Neuroticism, which has been previously identified as a risk factor, was also affirmed to confer significant risk for postpartum depression in a prospective study of 1,037 non-depressed pregnant women ^14^.

Efforts to identify pathophysiological cascades and associated biomarkers for perinatal mood disorders are ongoing ^15^; however, results have been heterogeneous to date and clear agreements have for the most part not emerged. However, low serum vitamin D stands out as a risk factor that has emerged across several recent studies ^16– 19^, suggesting that screening and supplementation for vitamin D deficiencies could be clinically important.

## Part II: Advances in screening for perinatal mood disorders

Recently, the considerable controversy regarding whether to recommend universal screening of all women for perinatal mood disorders has moved to a general consensus that this screening is worth the time and effort. The American College of Obstetricians and Gynecologists ^20^ and the American Academy of Pediatrics ^21^ published position statements recommending screening of all women for depression during pregnancy and postpartum. Most recently, the US Preventive Services Task Force updated guidelines for screening for depression in adults to include screening for perinatal depression ^22^. According to these recent guidelines, minimum screening for perinatal women includes screening for depression once during pregnancy and postpartum; however, since depression may evolve over the first postpartum year, it is recommended that clinicians consider continued screening, especially in high-risk groups. The recent findings of high rates of anxiety disorders in women who screen positive on the EPDS have increased the need for universal EPDS screening even further, since the EPDS has three questions (questions 3, 4, and 5) that can be used to identify anxiety symptoms, but further validation of the EPDS for screening of anxiety disorders is needed ^23^.

## Part III: Obstetrical, fetal, and developmental outcomes: maternal mental illness versus pharmacological exposures

It is difficult to distinguish those adverse effects on obstetrical, fetal, and developmental outcomes attributable to psychotropic medication from the adverse effects attributable to maternal mental illness. The infeasibility of conducting randomized trials in pregnant women makes studies in this field difficult to control and their results difficult to interpret. In earlier years, there was perhaps a bias on the part of researchers toward assuming that adverse outcomes were related to medication. However, several recent studies using careful designs targeted to disentangle the contributions of medication from those of maternal illness have further revealed the true scope of the effects of perinatal mental illness.

The emerging consensus from the existing literature is that although antenatal administration of serotonin reuptake inhibitor (SRI) antidepressants does carry risks, most notably reduced term of gestation, pulmonary hypertension of the newborn, and neonatal withdrawal, these risks are manageable and accrue largely to SSRI use in the third trimester ^24^. More importantly, they pale in comparison to the effects of maternal mental illness, which are still being articulated fully but include poorer fetal outcomes ^25^, long-term effects on child behavior ^26^ and cognition ^27^, and maternal self-harm, the last of which recently was again shown to be the most common cause of death in a population of perinatal women ^28^. Less information is available on perinatal use of other classes of psychotropics, including mood stabilizers and antipsychotics. Recent advances in these areas are summarized below.

### Obstetrical and fetal outcomes of antenatal exposure to maternal mental illness or psychotropic medication

An open question in the field is whether pharmacological treatment for maternal mental illness can mitigate the damaging effects of the mental illness. Venkatesh *et al.* ^29^ demonstrated that rates of preterm birth, an outcome that has previously been associated both with maternal depression and with antenatal SRI treatment, actually were indistinguishable from those of healthy controls in women whose depression was treated with SRIs though not in women with untreated depression.

A more recently revealed adverse effect of prenatal SRI treatment is an increased risk of postpartum hemorrhage ^30, 31^, although not all investigators have confirmed this ^32^. Nevertheless, the possibility of increased perinatal bleeding with antidepressants highlights the importance of considering anemia in the differential diagnosis of postpartum depression and the role for iron supplementation in this population ^33^.

In what is still the most comprehensive study to look at effects of maternal bipolarity, Boden *et al.* ^34^ carefully disaggregated the effects of mood-stabilizing medication (number treated = 320) from the effects of untreated bipolarity (number untreated = 554) and revealed that obstetric complications were increased in women with BD regardless of pharmacotherapy. Other studies have also addressed the issue of obstetric and perinatal effects of BD ^35^, but none has been able to address the distinctions between underlying BD and mood stabilizer effects in a larger population as Boden *et al.* ^34^ did.

The recent release of the first large, population-based study of antipsychotics in pregnancy (9,258 exposures) ^36^ echoes the findings of earlier, smaller studies of no systematic association with congenital malformations for most antipsychotics. There was a small possible increase in signal for cardiac malformations with risperidone, which has not been previously reported and would need confirmation with additional large studies. A finding of no increase in congenital malformations with prenatal antipsychotics was also published by the National Pregnancy Registry for Antipsychotics (303 exposures) ^37^.

Lin *et al.* ^38^ reported the results of a study of 696 mothers with schizophrenia. The authors found that antipsychotic treatment did not mitigate the increased risks for low birth weight and small size for gestational age that were associated with maternal schizophrenia, although they did find that first-generation antipsychotics were independently associated with preterm birth.

### Long-term outcomes of antenatal exposure to maternal mental illness or psychotropic medication

Many studies have looked at obstetrical and neonatal outcomes in maternal mental illness, but longer-term psychiatric and neurodevelopmental effects of antenatal exposure to maternal illness and to pharmacotherapy are an understudied area. Although many studies of this general question have been conducted ^39^, most have been small and poorly controlled for maternal illness. Thus, no true consensus has yet emerged, although several researchers have identified temporary delays in motor development as an area of potential concern ^40^.

Malm *et al.* ^41^ recently published a concerning finding of increased rates of depression, though not of several comparator psychiatric disorders, among adolescent children who had been prenatally exposed to selective serotonin reuptake inhibitors (SSRIs), and the rates were higher than those whose mothers had psychiatric disorders but no medication. Although this is only the first study to report such a finding in humans, the cohort was large (15,729 exposures), the design was rigorous, and the findings echo certain results from the animal literature ^42, 43^, but conflicting results are available from animal work as well ^44^. Thus, this particular outcome, which otherwise has not been at all well investigated in humans, is one that should be kept in mind for additional large, well-designed prospective trials.

A population-based study ^45^ of 20,183 children included 183 siblings discordant for prenatal antidepressant exposure. Some information was available on maternal diagnosis, symptom severity, and drug exposure window. Among six subtypes of internalizing and externalizing behaviors, anxiety showed a moderate association with antenatal SRI exposure at 36 months but not at 18 months of age. No other assessed outcomes were associated with SRI exposure at either 18 or 36 months in adjusted analyses. Mother’s history of depression was affirmed to be predictive of child internalizing behaviors.

A well-designed study of 45 sibling pairs who differed in exposure to SRIs found no effect of medications on IQ or behavior at 3 to 6 years, although maternal depression was again affirmed to be of major importance in predicting child behavior problems ^26^. This study included extensive information on maternal diagnosis, symptom severity, and window of drug exposure.

Malm *et al.* ^41^ published an analysis of cognitive outcomes after antenatal SSRI exposure from national registry data that included 384 antenatal SSRI exposures among 51,404 singleton pregnancies. This group observed a small increase in rates of speech/language disorder among 3-year-olds with SSRI exposure. The magnitude of the finding (95% confidence interval (CI) 1.26–1.86) was small and crucially was also present as a difference between the unmedicated psychiatric disorder versus healthy control group (95% CI 1.03–1.58), suggesting a role for maternal mental illness in this outcome. It is relevant that while the authors had an unmedicated, psychiatrically ill comparator group and were able to adjust statistically for symptoms of maternal anxiety and depression, unmeasured differences between groups are likely to play a major role in this type of outcome. Other investigators have managed this problem by using, for example, sibling comparisons ^26^, or comparisons with other psychiatric outcomes, and studies using this type of comparison are more informative than those such as the one by Malm *et al.*, ^41^ who did not address unmeasured differences between study groups.

An area of ongoing investigation involves the attempt to associate prenatal SSRI exposure with autism spectrum outcomes; however, the gold standard in this field remains the study by Sørensen *et al.* ^46^, who found no association of prenatal SSRIs with autism that survived sibling analysis. Multiple studies asserting such a link have been published before and since but none with the crucial control for unmeasured differences that was provided by the sibling analysis.

Baker *et al.* recently released the six-year follow-up of their ongoing study of children exposed antenatally to anticonvulsants ^47^. Persistent cognitive deficits were associated with antenatal exposure to valproic acid, as at earlier time points. Results for lamotrigine and carbamazepine remained reassuring.

Very few studies are available of neurodevelopmental outcomes after antenatal antipsychotic exposure. A re-analysis of previously published data ^48^ on 63 exposed infants uncovered an apparent increased risk for neonatal adaptation in infants exposed to clozapine versus other atypical antipsychotics ^49^. As with the other available studies in this area ^48, 50^, cohorts were small and it was impossible to control adequately for the effects of maternal mental illness. 

## Part IV: Recent advances in acute pharmacologic and psychotherapy treatment of perinatal mood disorders

A meta-analysis of 40 randomized studies of cognitive behavior therapy (CBT) during pregnancy and the first year postpartum reported that CBT was associated with significant reduction in depressive symptoms ^51^. Recent advances in CBT delivery have included internet-based CBT for postpartum depression, and this method was associated with improvement in depressive symptoms in preliminary investigations ^52^. Interpersonal psychotherapy (IPT) remains an effective evidence-based treatment for postpartum depression ^53^, and recent adaptations have included a pilot study of partner-assisted IPT ^54^ and mother-infant IPT ^55^, both of which appear promising.

Although several systematic reviews have been completed that report that antidepressants are associated with significant reductions in depressive symptoms in women with unipolar depression postpartum ^56, 57^, there is only one published study to date of the acute treatment of acute bipolar postpartum depression ^58^. In a chart review of 18 bipolar women who were treated with quietapine alone (or with sedative hypnotic), Sharma *et al.* ^58^ reported that 83% were much improved or improved on retrospective Clinical Global Impression Scale scores. Given that recent investigations have demonstrated high rates of postpartum depression in patients with BD, future psychopharmacologic and psychotherapeutic treatment studies are needed in this population.

## Part V: Advances in preventing perinatal mood disorders

Although there have been many advancements in the study of risk factors for perinatal depression, advancement in methods to prevent perinatal depression has remained less studied, except in high-risk groups, such as women with BD. Recent studies have built upon the initial reports of Viguera *et al.* ^4^ of high rates of relapse in bipolar pregnant women who discontinue lithium during pregnancy. For instance, Rosso *et al.* ^59^ reported in a study of 17 women with lithium-responsive bipolar I that continued use of lithium in pregnancy and postpartum resulted in reduced rates of postpartum relapse compared with previously published unmedicated relapse rates of 66% ^60^ to 75% ^61^ in this population. Uguz *et al.* ^62^ reported the first small case series of relapse risk in women who switched from other mood stabilizers such as lithium or valproic acid prior to pregnancy to olanzapine or quietapine in pregnancy. In their sample of eight women, none experienced relapse. Sharma and Sommerdyk ^63^, in their report of prevention of postpartum depression in three women with bipolar II disorder with the use of lamotrigine, also highlight the need for larger randomized trials of mood stabilizer treatments for management of BD in pregnancy and postpartum. Lithium prophylaxis immediately postpartum has been shown to decrease relapse in those very-high-risk women with histories of postpartum mania and psychosis ^64^.

## Summary and conclusions

The most recent updates in the field of perinatal mental health highlight the importance of risk factors such as BDs, anxiety disorders, attachment insecurity, and neuroticism. This closer examination of factors that relate to individual personality traits represents an evolution from an earlier period in which unipolar history and social stressors were more heavily emphasized. The new emphasis on personality traits holds the promise for more accurate, individually tailored risk assessments by clinicians.

At the same time, recognition of the importance of early detection has been growing, as underlined by the recent release of guidelines from several bodies of experts who recommend increased attention to careful and regular screening for mood disorders and comorbid anxiety disorders in perinatal women. Although “universal screening” recommendations represent an advancement in early recognition and treatment, more research on susceptibility factors is needed so that high-risk groups can be easily identified through targeted screening practices.

There have been several important additions to the evolving conversation about the relative contributions of maternal mental illness versus psychotropic medication to obstetric, neonatal, and childhood outcomes. On the whole, these highlight the morbidity associated with maternal mental illness to a greater degree than has been previously recognized. The question of whether medication can mitigate these effects is still an open one. Recent work suggests that this may be true for at least some outcomes with respect to the use of SRI antidepressants for depression or anxiety but may not be true for the use of mood stabilizers or antipsychotics in women with BD.

For future research, questions remain regarding the respective roles of maternal mental illness and psychiatric medications in obstetrical and infant outcomes, and future studies must control for frequently unmeasured differences between groups, such as confounding by intention, family history of psychiatric illness, and developmental disorders. Larger and more carefully controlled studies of long-term neurodevelopmental outcomes in children are also lacking, particularly so for mood stabilizers and antipsychotics. Another unanswered question concerns the potential utility of tapering or briefly discontinuing antidepressants toward the end of pregnancy, as the most well-established adverse effects of medication (reduced term of gestation, pulmonary hypertension of the newborn, and neonatal adaptation) are all associated with third-trimester exposure. So far, data to inform this approach are lacking.
